# Navigating Treatment Dilemmas: Recalcitrant Pemphigus and the Burden of Multiple Comorbidities

**DOI:** 10.7759/cureus.56357

**Published:** 2024-03-18

**Authors:** Priya Garg, Kshitiz Lakhey, Nishtha Mishra, Yash Buccha, Kirti S Deo

**Affiliations:** 1 Dermatology, Dr. D. Y. Patil Medical College, Hospital and Research Centre, Pune, IND; 2 Dermatology, Venereology, and Leprosy, Dr. D. Y. Patil Medical College, Hospital and Research Centre, Pune, IND

**Keywords:** deep vein thrombosis, hydrogen peroxide (h2o2), hyaluronidase, mycophenolate mofetil, plasmapheresis, recalcitrant pemphigus

## Abstract

Pemphigus vulgaris is a chronic autoimmune disease of the skin caused by the production of autoantibodies targeting desmogleins 1 and 3 usually presenting in individuals with an average age of onset of approximately 40 years. A 35-year-old obese, diabetic woman presented with fluid-filled lesions over her body for three months along with erosions and painful ulcers in her mouth and genital area for two months. Based on clinical and histopathological studies, the patient was diagnosed as a case of pemphigus vulgaris. She was started on conventional treatment with oral corticosteroids followed by pulse therapy and mycophenolate mofetil. Rituximab infusion was scheduled but could not be administered due to elevated D-dimer values. The patient underwent screening for deep vein thrombosis (DVT) and received subcutaneous enoxaparin and oral rivaroxaban. She developed severe sepsis for which she was treated with systemic antibiotics. She subsequently developed acute renal failure and underwent hemodialysis. The patient's clinical condition further deteriorated, which necessitated therapeutic plasma exchange (TPE). Collagen, colloidal silver, and silicone foam dressings were done to hasten wound healing. Two distinct approaches were employed to eliminate the pseudomembrane on the wounds. One portion was treated with hydrogen peroxide (H_2_O_2_), while the other was with hyaluronidase. The hyaluronidase treatment resulted in considerable improvement of the lesions. Intravenous immunoglobulin (IVIG) infusion was scheduled. However, the treatment could not be administered as the patient succumbed to death due to pulmonary thromboembolism (PTE) secondary to DVT.

## Introduction

Pemphigus vulgaris (PV) is an autoimmune condition characterized by the formation of blisters within the skin and erosions in the mucosa. It is characterized by the presence of immunoglobulin G (IgG) class autoantibodies against the intercellular adhesion molecules, specifically desmogleins 3 and 1, which leads to the loss of cell adhesion and formation of intraepidermal clefts [1]. The main objectives of treatment are to reduce blister formation, avoid infections, facilitate healing of erosions, and achieve long-lasting remission. Superimposed infections, facilitated by the breakdown of the protective barrier normally provided by skin or immunosuppression secondary to treatment, frequently contribute to morbidity and mortality in patients with PV [2]. Breakthroughs in corticosteroid and steroid-sparing therapies along with wound care in recent years have significantly improved the outlook for PV in recent years [3]. 

## Case presentation

**Figure 1:**
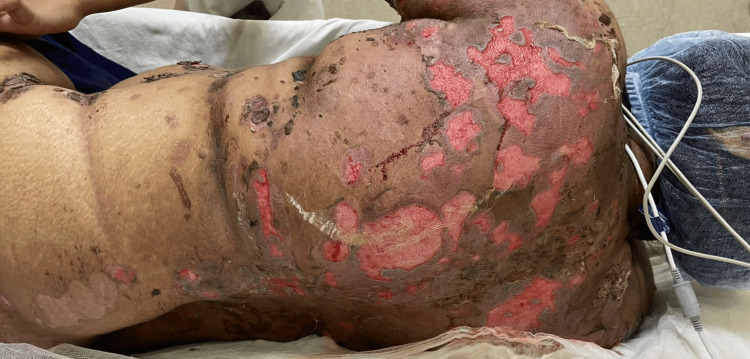
Multiple erosions and crusts present over the back.

**Figure 2:**
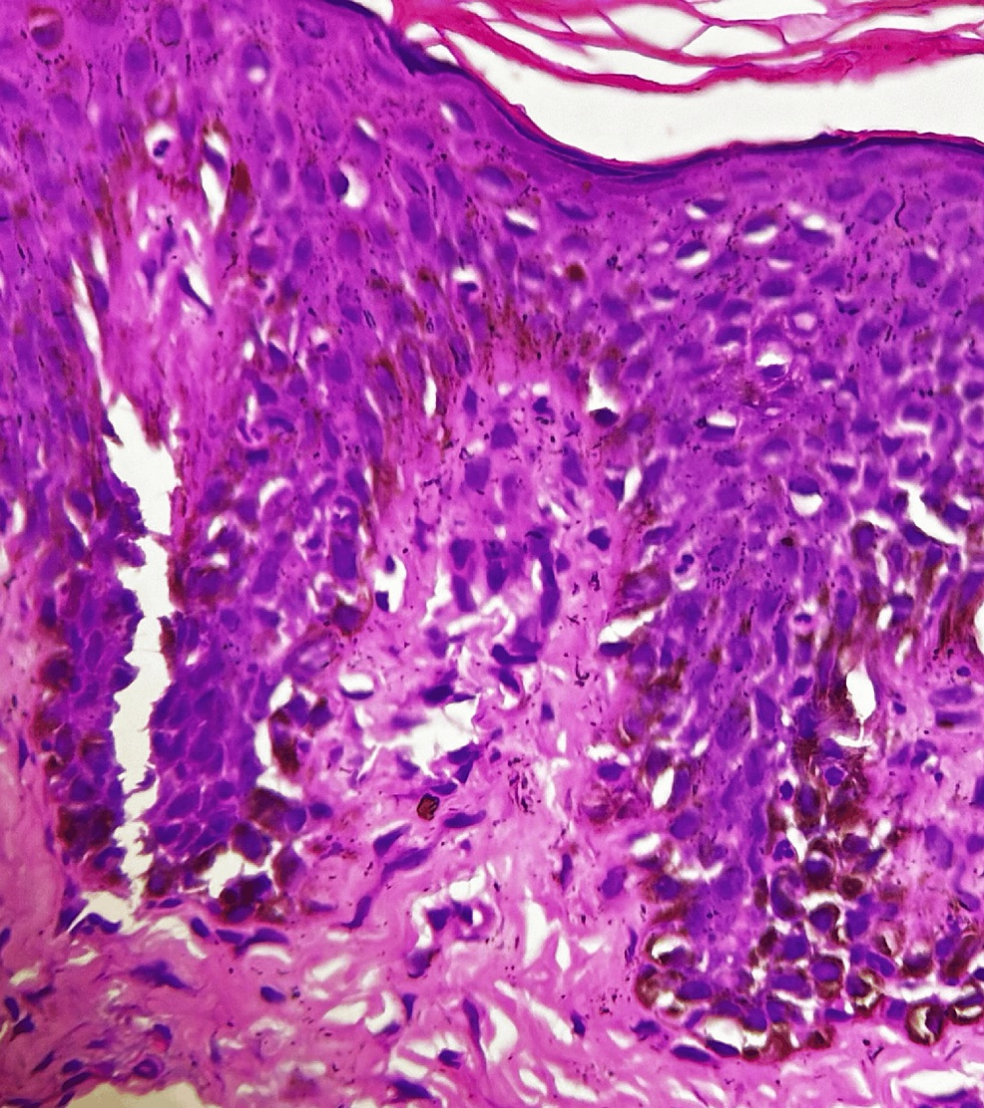
Histopathological examination of a lesional punch biopsy showed a suprabasal split with predominant neutrophils and a few eosinophils.

**Figure 3:**
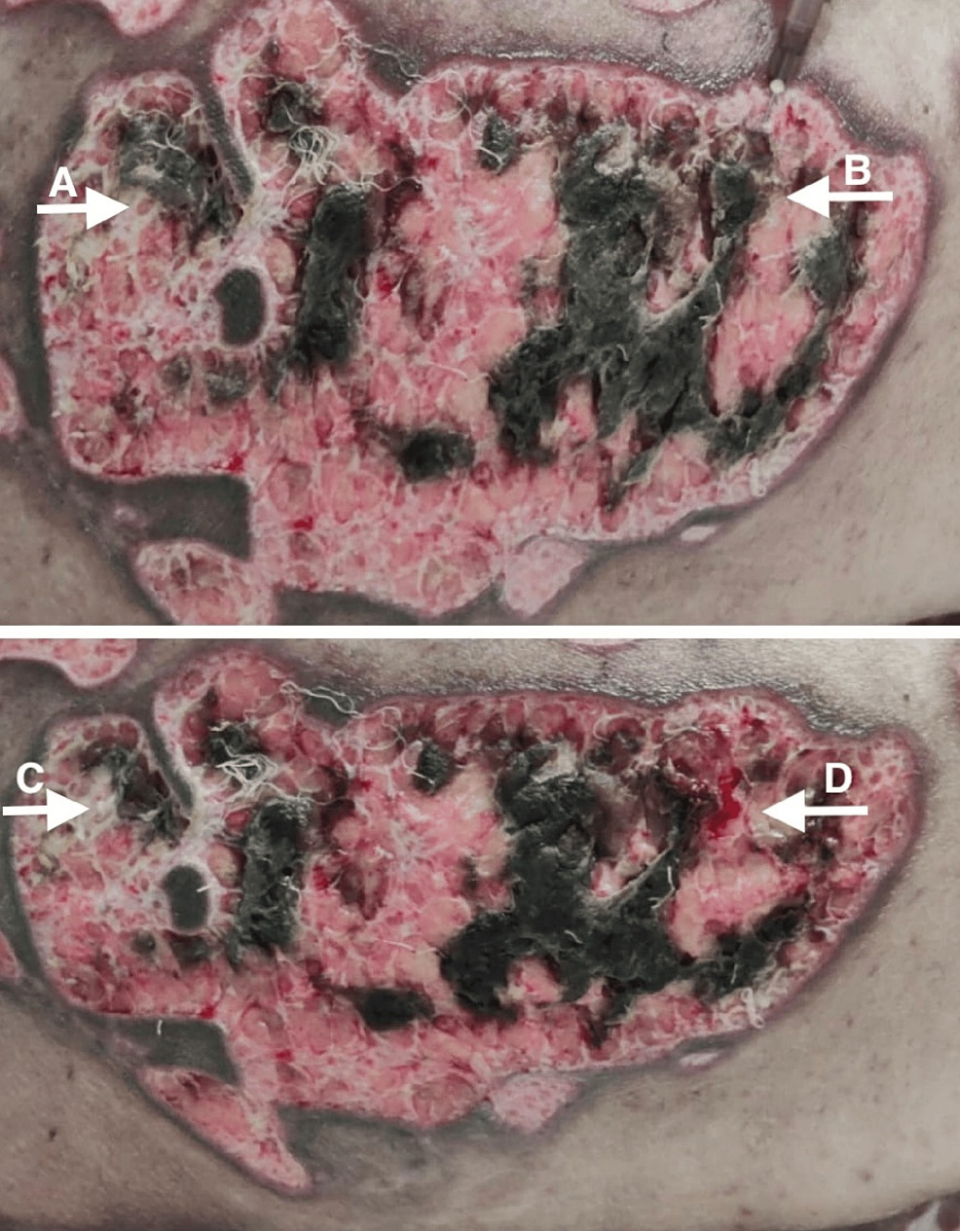
Eschar before and two days after treatment with 6% H2O2 and hyaluronidase. (A) Eschar before being treated with H_2_O_2_; (B) eschar before being treated with hyaluronidase; (C) eschar after treatment with 6% H_2_O_2_; (D) eschar after treatment with hyaluronidase. H_2_O_2_, hydrogen peroxide

A 35-year-old, overweight woman presented with multiple erythematous plaques with crusting and erosions on the abdomen, chest, back, and bilateral upper arms for three months. Additionally, the patient had ulcers over her oral mucosa and genital area, along with scalp involvement for two months (Figure 1). The pemphigus disease area index (PDAI) was 30, suggesting a severe level of disease activity. There was no involvement of the ocular mucosa, nails, palms, and soles. The patient had a body mass index (BMI) of 28 kg/m^2^. She had a known case of hypothyroidism for five years and diabetes mellitus for the past two months, which was controlled by oral hypoglycemic agents. PV was clinically diagnosed and then verified through immunofluorescence and histological investigations. The enzyme-linked immunosorbent assay (ELISA) analysis of IgG anti-Dsg1 and anti-Dsg3 yielded titers of 101.50 and 210 RU/mL, respectively. Histopathological examination of a lesional punch biopsy showed a suprabasal split with predominant neutrophils and a few eosinophils (Figure 2). Intercellular IgG and C3 deposits were seen on direct immunofluorescence. The patient was initially started on oral corticosteroids (0.5-1.0 mg/kg/day) but was shifted to dexamethasone pulse therapy and was subsequently discharged on mycophenolate mofetil with a maintenance dose of oral steroids. However, she was not compliant with her medications and followed up after one month with a worsened clinical condition. Intravenous dexamethasone was administered, and oral mycophenolate mofetil was restarted. Due to the lack of improvement in the healing of the lesions, collagen dressings, and colloidal silver ointment were used on the eschar, along with absorbent foam dressings. Two methods were used to remove the wound pseudomembrane. Hydrogen peroxide (H_2_O_2_) was used to treat one portion of the substance, while hyaluronidase was used to treat the other (Figure 3). The lesions improved greatly after hyaluronidase treatment. Her serum electrolytes, blood sugar, and D-dimer levels were deranged. Venous duplex ultrasound demonstrated thrombosis in the bilateral internal jugular vein and femoral vein for which she was started on injection of enoxaparin and oral rivaroxaban. Pus culture and urine culture revealed growth of methicillin-resistant Staphylococcus aureus and Klebsiella pneumoniae, respectively. She was given systemic antibiotics for the same (meropenam, linezolid, and then later tigecycline according to pus culture and sensitivity reports). The patient then complained of decreased urine output. On investigations, there was increased serum urea and creatinine levels. A stat dose of intravenous furosemide was administered, and a bladder flush was performed. Furthermore, therapeutic plasma exchange (TPE) was performed to ameliorate the clinical manifestations. The patient finally became vitally stable. In addition, neither blood nor pus culture showed the growth of the organism. Administration of intravenous Igs was planned. However, the patient suddenly developed pulmonary thromboembolism, which unfortunately led to her demise.

## Discussion

PV is an autoimmune skin and mucosal condition characterized by flaccid blisters and erosions, caused by autoantibodies, which target desmogleins 1, 3, 6, and 7 found in the keratinocytes [4]. The present-day approach to treat PV involves the use of systemic glucocorticosteroids, immunosuppressive medications such as azathioprine, mycophenolate mofetil, or cyclophosphamide and monoclonal antibodies such as rituximab [5]. Other treatment modalities include intravenous immunoglobulin (IVIG), immunoadsorption, and TPE [6]. Recalcitrant or resistant pemphigus is characterized by recurrent blister formation despite immunosuppressive therapy with corticosteroids >10 mg/day of tab prednisolone or an equivalent level of immunosuppression [1]. Factors affecting disease activity and predicting recalcitrant pemphigus risk include the body surface area involved, average steroid dose for disease control, earlier age of onset, comorbidities such as obesity, diabetes, hypertension, dyslipidemia, and other autoimmune diseases, as well as the duration of the disease [1].

The second cycle of dexamethasone pulse therapy could not be administered as the patient had documented sepsis, uncontrolled blood sugar levels, and extensive venous thrombosis. Furthermore, the patient refused administration of cyclophosphamide due to its known side effects of decreased ovarian reserve and premature gonadal failure and she wanted to conceive in the future [7]. Similarly, rituximab could not be administered as it is contraindicated in cases of sepsis [8]. TPE is performed in patients with severe PV who show little or no response to conventional steroid treatment. It removes molecules with high molecular weight, such as pathogenic autoantibodies, immune complexes, cryoglobulins, and toxins, within the plasma [9]. Though it was used as an adjunct to improve the renal complications, it also helped in the improvement of the lesions by the aforementioned mechanism.

Wound care plays a significant role in the management of pemphigus. Antimicrobial dressings, such as those containing iodine and silver, are frequently employed in the treatment of wounds that are susceptible to infection [2]. H_2_O_2_ is needed for wound healing, microbial removal, and hemostasis [10]. Hyaluronidase-treated wounds produce more granulation tissue, reduce edema, and regulate the inflammatory response by modifying pro- and anti-inflammatory cytokines, growth factors, and eicosanoid mediators. In addition, it boosts gene expression of PPAR γ and β/δ, collagen content during early healing, and angiogenesis [11]. Therefore, hyaluronidase may offer a notable advantage compared to other approaches in the therapy of wounds.

## Conclusions

In this case, the poor prognosis can be attributed to several factors, including an earlier age of onset, noncompliance with the treatment regimen, and the presence of comorbidities such as obesity, diabetes, and hypothyroidism. The nonhealing cutaneous lesions of recalcitrant pemphigus required vigorous treatment with immunosuppressants. However, the lesions themselves predisposed the patient to infections and sepsis. This dilemma highlights the importance of proper wound care and the need for further advances in alternative therapies for controlling the disease activity, which can be used even when immunosuppression is contraindicated.
